# Supplementary material for: Structure and lipid dynamics in the maintenance of lipid asymmetry inner membrane complex of A. baumannii
Source: Commun Biol. 2021 Jun 29;4:817. doi: 10.1038/s42003-021-02318-4 (PMC8241846; doi:10.1038/s42003-021-02318-4)
Supplement: Supplementary file 2 — Supplementary material. [file 42003_2021_2318_MOESM2_ESM.pdf]

Supplementary Information

Structure and lipid dynamics in the *A. baumannii* maintenance of lipid asymmetry inner membrane complex

Daniel Mann[1,&], Junping Fan[2,%*], Kamolrat Somboon[3*], Daniel P. Farrell[4], Andrew Muenks[4], Svetomir B. Tzokov[1], Frank DiMaio[4], Syma Khalid[3], Samuel I. Miller[2,4,5], Julien R. C. Bergeron[1,6*]

[1] Department of Molecular Biology and Biotechnology, The University of Sheffield, Sheffield, United Kingdom

[2] Department of Microbiology, The University of Washington, Seattle, USA

[3] Department of Chemistry, University of Southampton, Southampton, UK

[4] Department of Biochemistry, The University of Washington, Seattle, USA

[5] Department of Genetics, The University of Washington, Seattle, USA

[6] Randall Division of Cell and Molecular Biophysics, King's College London, London, UK

[&] Current address: Ernst-Ruska-Centre 3, Forschungszentrum Jülich, Germany

[%] Current address: Department of Pharmacology, The University of Washington, Seattle, USA

* These authors have contributed equally to this work

*Correspondence to: julien.bergeron@kcl.ac.uk

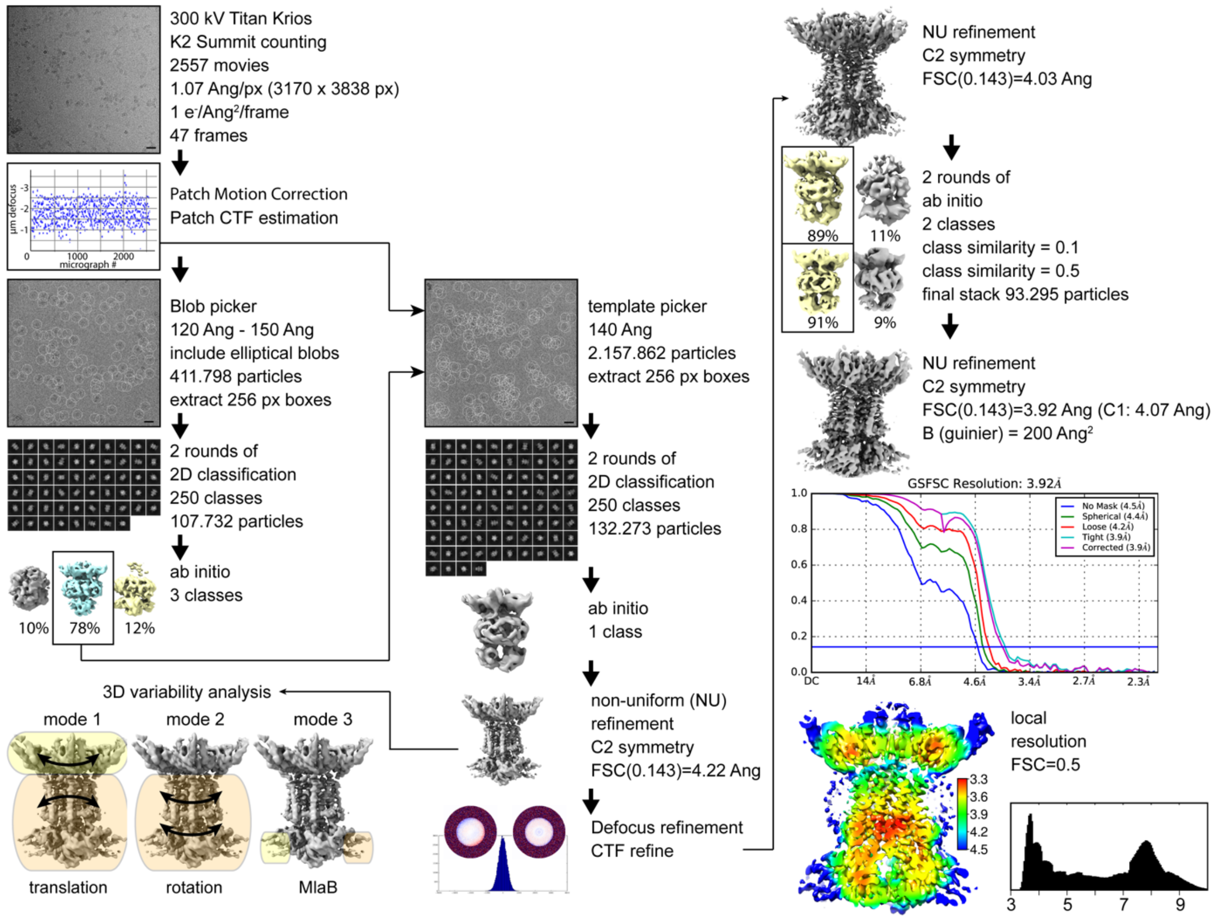

Supplementary Figure 1: processing of the MlaBDEF-AppNHp dataset in CryoSPARC v2.14.2. 2557 micrographs (scale bar: 140 Å) were recorded at 300 kV on a Titan Krios with an energy filtered Gatan BioQuantum967 detector (K2 summit) with a Nyquist frequency of 2.14 Å. Patch motion correction resulted in a detected defocus range of -1 to -2.5 µm. Initial blob picking with a diameter range from 120-150 Å including elliptical shapes were extracted and 2D classified two times into each 250 classes. Ab initio 3D models were generated and the best class was used to re-pick particles template-based. After two rounds of 2D classification an ab initio 3D model was generated and refined using CryoSPARCs Non-Uniform (NU) refinement procedure with C2 symmetry, followed by global and local CTF refinement and two rounds of ab initio model generation with class similarity values of 0.1 and 0.5, respectively. The final particle stack contained 93.295 particles and was NU refined to 3.92 Å with C2 symmetry (4.07 Å with C1 symmetry). The map was sharpened with the Guinier plot B-factor of -200 Å^2. Fourier Shell Correlation plot is indicated as well as local resolutions at FSC=0.5 projected on the final map. A histogram with the full local resolution range (from 3.3 Å in red to 4.5 Å in blue) is also indicated. The first high resolution 3D structure was used as an input for CryoSPARCs 3D variability jobtype

with 6 modes. Only the first three modes showed global changes; firstly, translation of MlaD against MlaBEF (Supplemental Movie 1), secondly, rotation of the MlaBEF part against MlaD (Supplemental Movie 2) and thirdly, alternating appearance of MlaB, indicating lower occupancy of this part compared to the MlaDEF part.

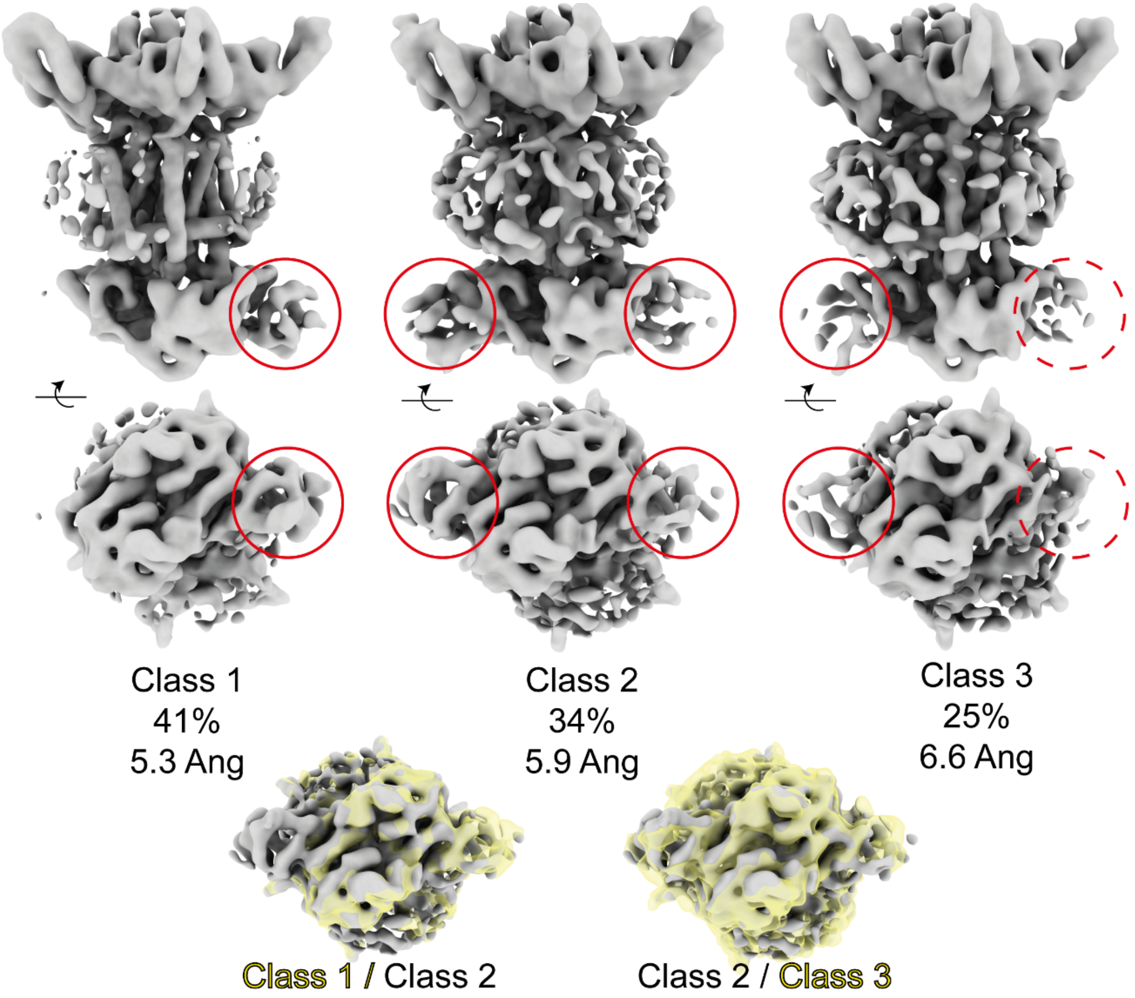

Supplementary Figure 2: MlaB binding (red circles) occurred on both binding sites in about 50% of the particles (classes 2 and 3) and on only one binding site in the other 50% of the particles (class 1). Maps were obtained by Non-Uniform refinement in C1 symmetry after heterogeneous refinement with 3 classes in CryoSPARC. Alignments of Classes 1-3 snow no major structural changes upon MlaB binding.

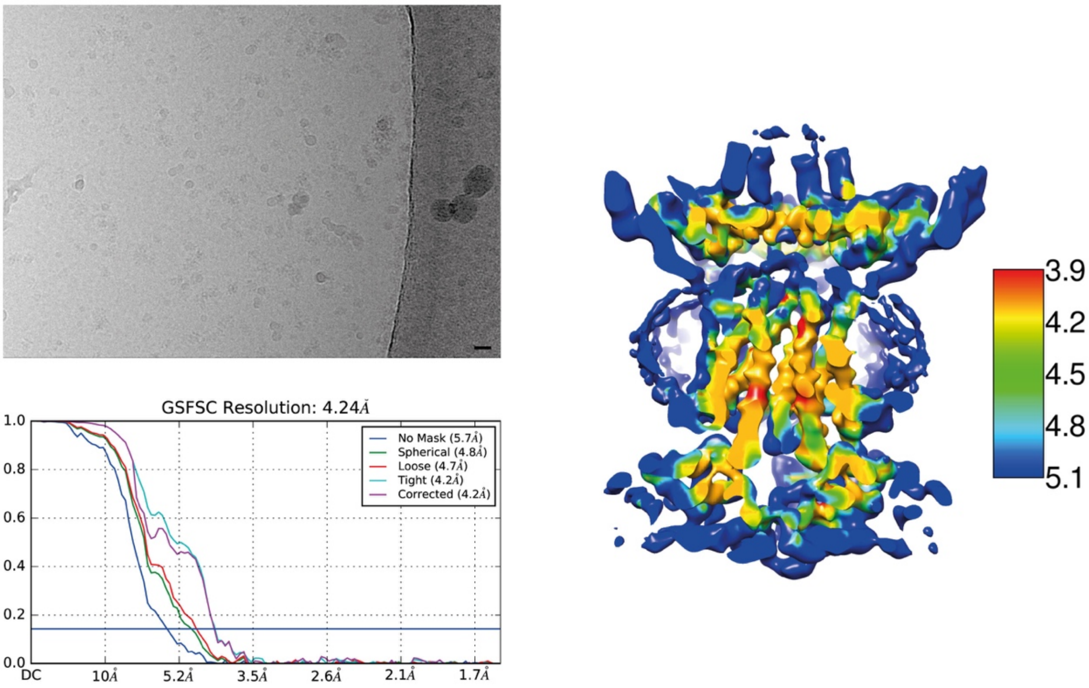

Supplementary Figure 3: processing of the apo MlaBDEF$_{ab}$ dataset in CryoSPARC v2.14.2. Micrographs (Scale bar: 140 Å) were recorded on 300 kV Titan Krios instruments equipped with Gatan K3 Bioquantum detector in counting mode. After blob picking and 2D classification selected 2D classes were used for template picking. After several rounds of ab initio 3D structure generation and 3D classification, non-uniform refinement with C2 symmetry led to a map with a global resolution of ~ 4.2 Å. The map colored by local resolution is shown, from 3.9 Å in red to 5.1 Å in blue.

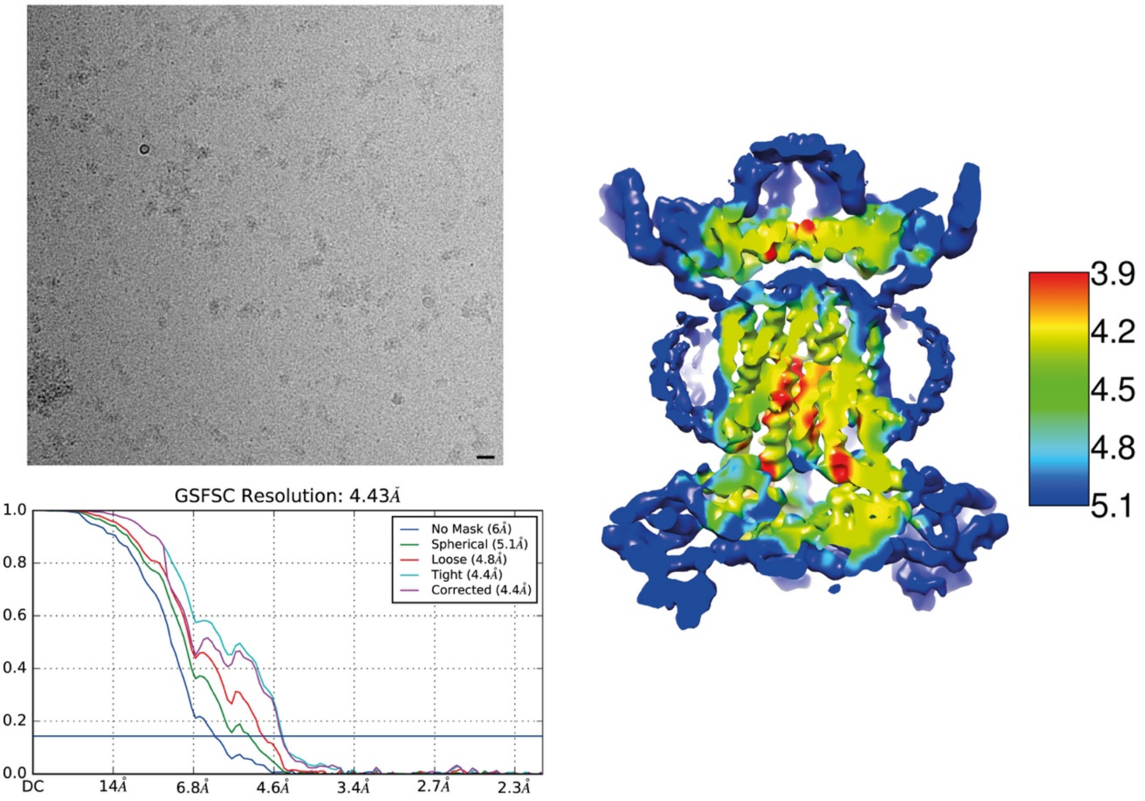

Supplementary Figure 4: processing of the MlaBDEF$_{ab}$ -ADP dataset in CryoSPARC v2.14.2. Micrographs (scale bar 140 Å) were recorded on 300 kV Titan Krios instruments equipped with Gatan K2 Summit detector in counting mode. After blob picking and 2D classification selected 2D classes were used for template picking. After several rounds of ab initio 3D structure generation and 3D classification, non-uniform refinement with C2 symmetry led to a map with a global resolution of ~ 4.4 Å. The map colored by local resolution is shown, from 3.9 Å in red to 5.1 Å in blue.

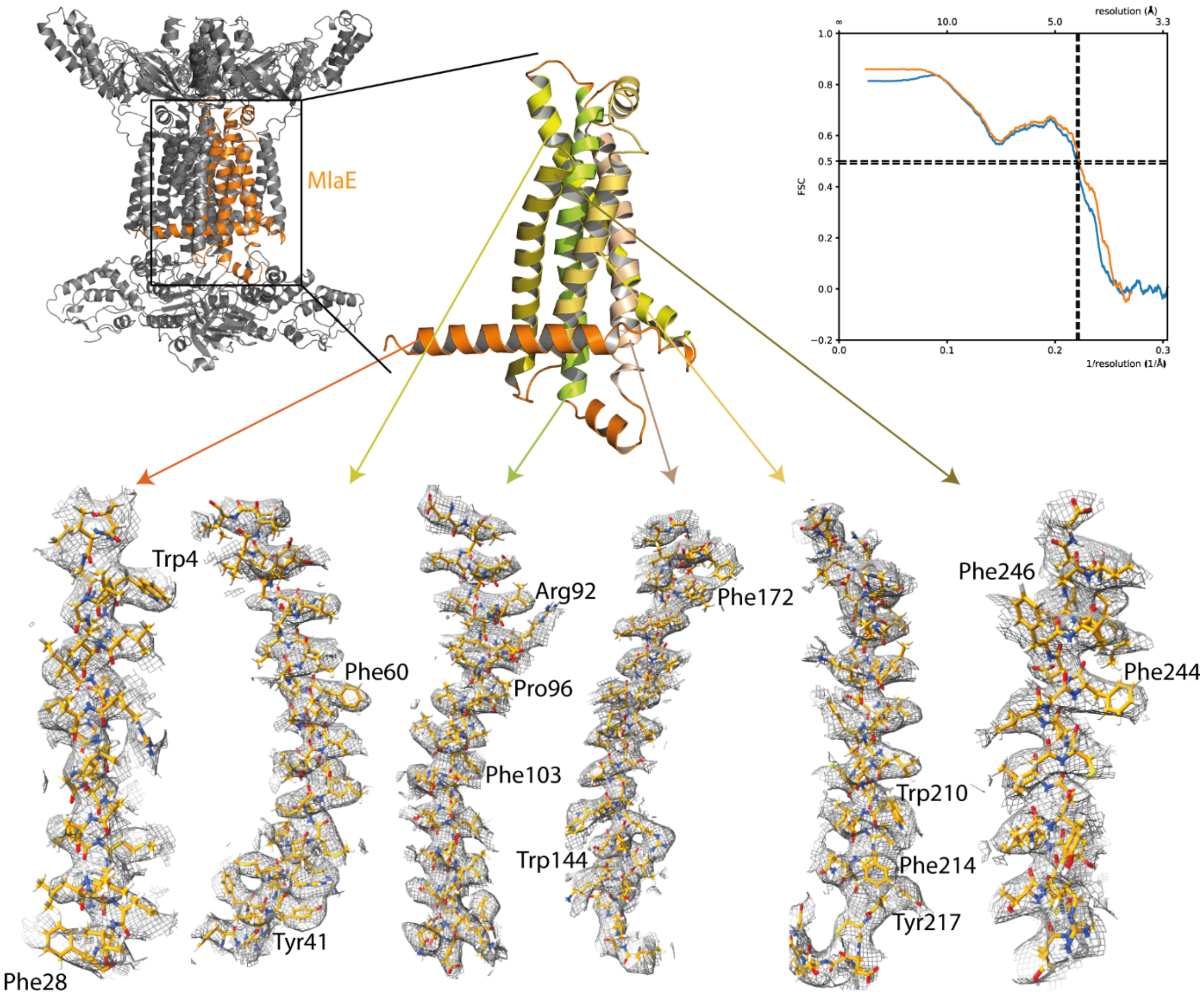

Supplementary Figure 5: de novo model building of MlaE (orange). Large side chains that allowed sequence mapping are indicated as well as map-to-model FSC of the whole $MlaBDEF_{ab}$ protein complex.

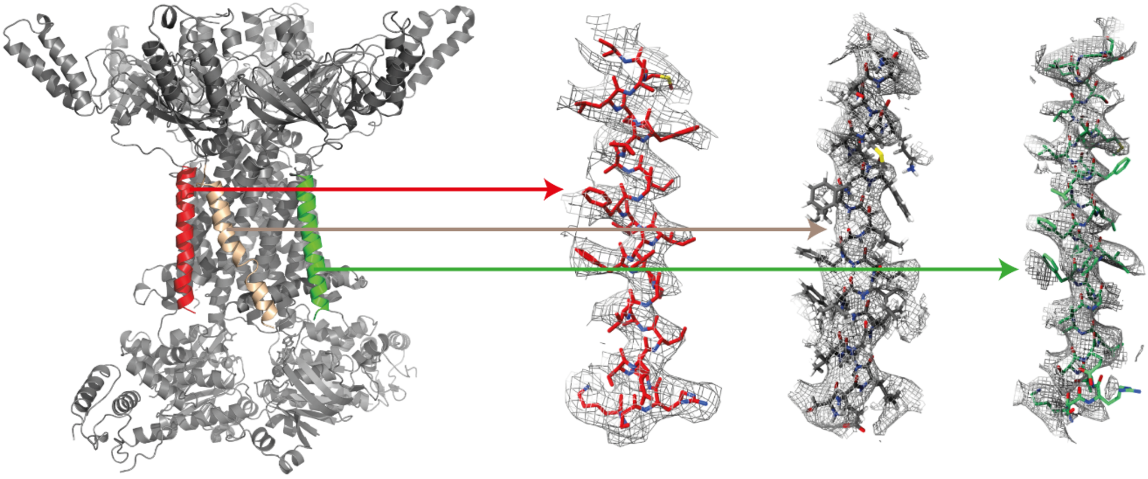

Supplementary Figure 6: Enclosed N-Helix of MlaD is significantly better resolved compared to peripheral MlaD N-helices (grey, green).

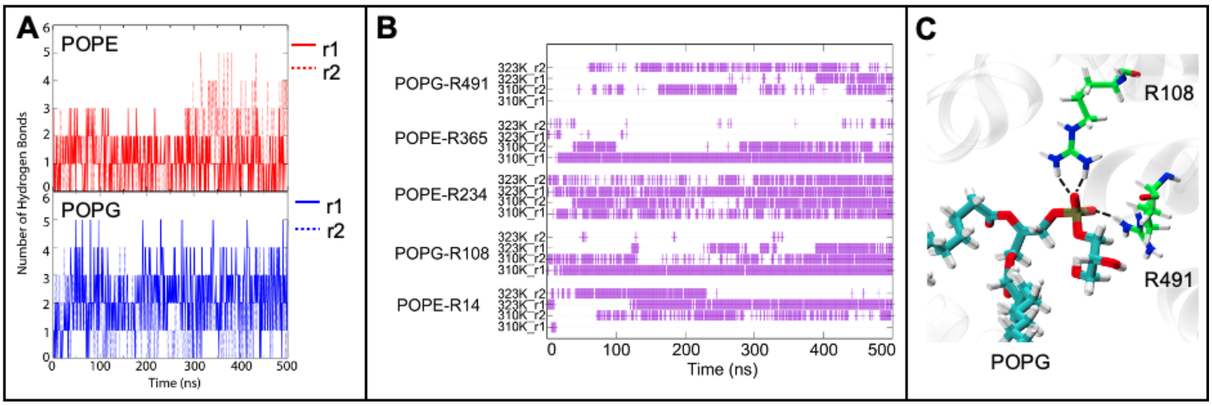

Supplementary Figure 7: Lipid dynamics in the cytosolic binding pockets. a: Number of hydrogen bonds as a function of time, between the two lipids in the binding site and the protein, where r1 and r2 are the two independent simulations at 310 K. b: Contact matrix of hydrogen bonds between lipid and protein in the four simulations. c: Example of POPC binding via its phosphate group with two arginines simultaneously.

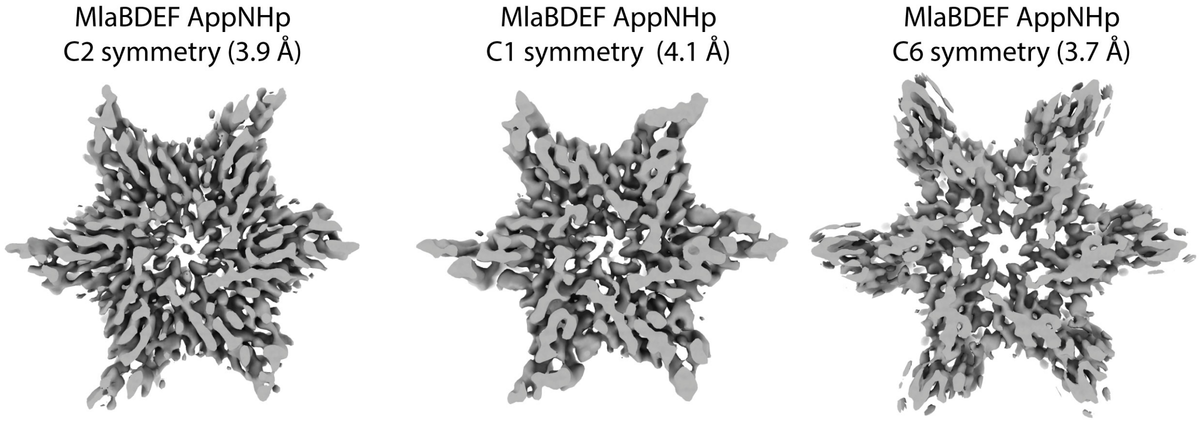

Supplementary Figure 8: MlaD crown region of MlaBDEF-AppNHp with C2 symmetry, with no symmetry or with C6 symmetry applied during reconstruction. The central detergent molecule as well as the six peripheral detergent molecules are clearly visible in all applied symmetries. C6 symmetry does not help with map interpretation of MlaD, as the periphery gains symmetry artifacts.

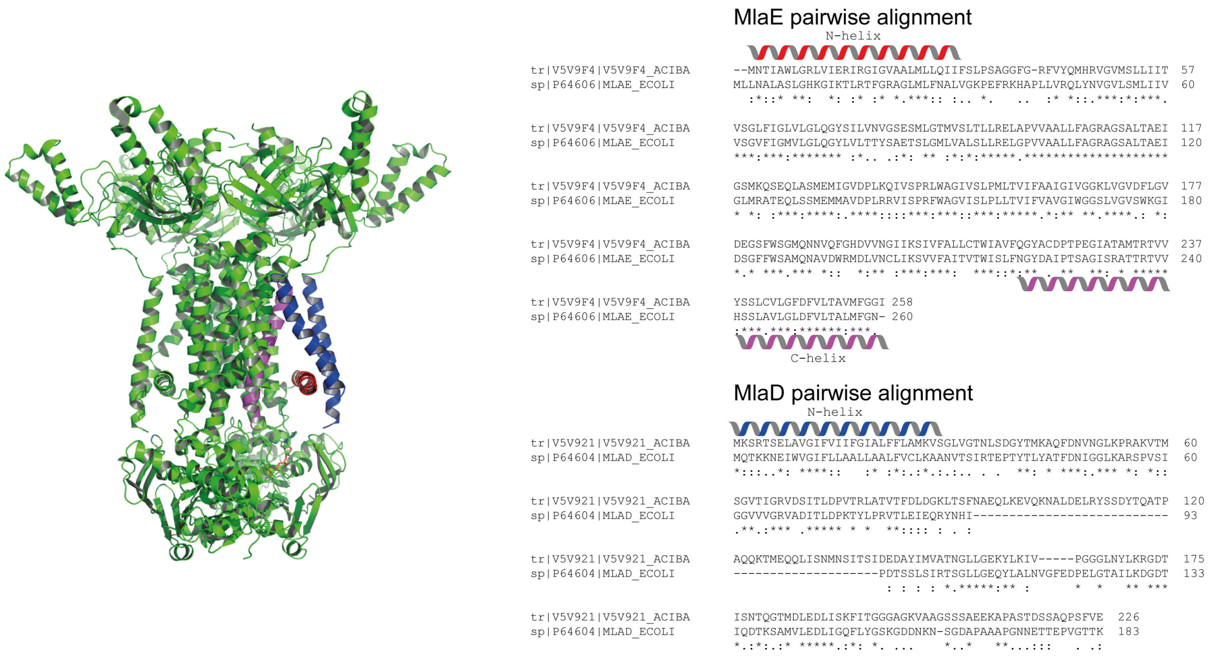

Supplementary Figure 9: Sequence identity between *Acinetobacter baumannii* MlaE and MlaD as well as *Escherichia coli* MlaE and MlaD in the proposed cytosolic lipid binding pocket. The pocket constitutes mainly of the N-terminal helix of MlaE (red), the C-terminal helix of MlaE (magenta) and the N-terminal helix of MlaD. The C-terminal helix of MlaE shows highest sequence identity, while MlaD and MlaE N-helices differ more. Pairwise alignment was created using ClustalOmega (www.ebi.ac.uk) with default protein parameters.

Supplementary Table 1: Equilibrium Protocol

Step	Time Step (fs)	Total Time (ns)	Protein Backbone Restraints (kJ mol^{-1} nm $^{-2}$)	Protein Sidechain Restraints (kJ mol^{-1} nm $^{-2}$)	Ensemble
1	1	0.125	4000	2000	NVT
2	1	0.125	2000	1000	NVT
3	1	0.125	1000	500	NPT
4	2	0.5	500	200	NPT
5	2	0.5	200	50	NPT
6	2	20	50	0	NPT

Supplementary Table 2: Summary of the equilibrium MD simulation systems

System	Substrate	Temperature (K)	Simulation Length (ns)
Apo Mla	-	310	500 ($\times$ 2)
Apo Mla	-	323	500 ($\times$ 2)
Holo Mla	7 POPE	310	500 ($\times$ 2)
