# Supplementary material for: Structure and lipid dynamics in the maintenance of lipid asymmetry inner membrane complex of A. baumannii
Source: Commun Biol. 2021 Jun 29;4:817. doi: 10.1038/s42003-021-02318-4 (PMC8241846; doi:10.1038/s42003-021-02318-4)
Supplement: Supplementary file 3 — Description of Supplementary Files. [file 42003_2021_2318_MOESM3_ESM.pdf]

Description of Additional Supplementary Files

File name: Supplementary data 1

Description: The raw data underlying the graph in Supp. Fig. 1

File name: Supplementary data 2

Description: The raw data underlying the graph in Supp. Fig. 3

File name: Supplementary data 3

Description: The raw data underlying the graph in Supp. Fig. 4
